# Supplementary figures and images for: Key Amino Acids for Transferase Activity of GDSL Lipases
Source: Int J Mol Sci. 2022 Dec 1;23(23):15141. doi: 10.3390/ijms232315141 (PMC9736205; doi:10.3390/ijms232315141)

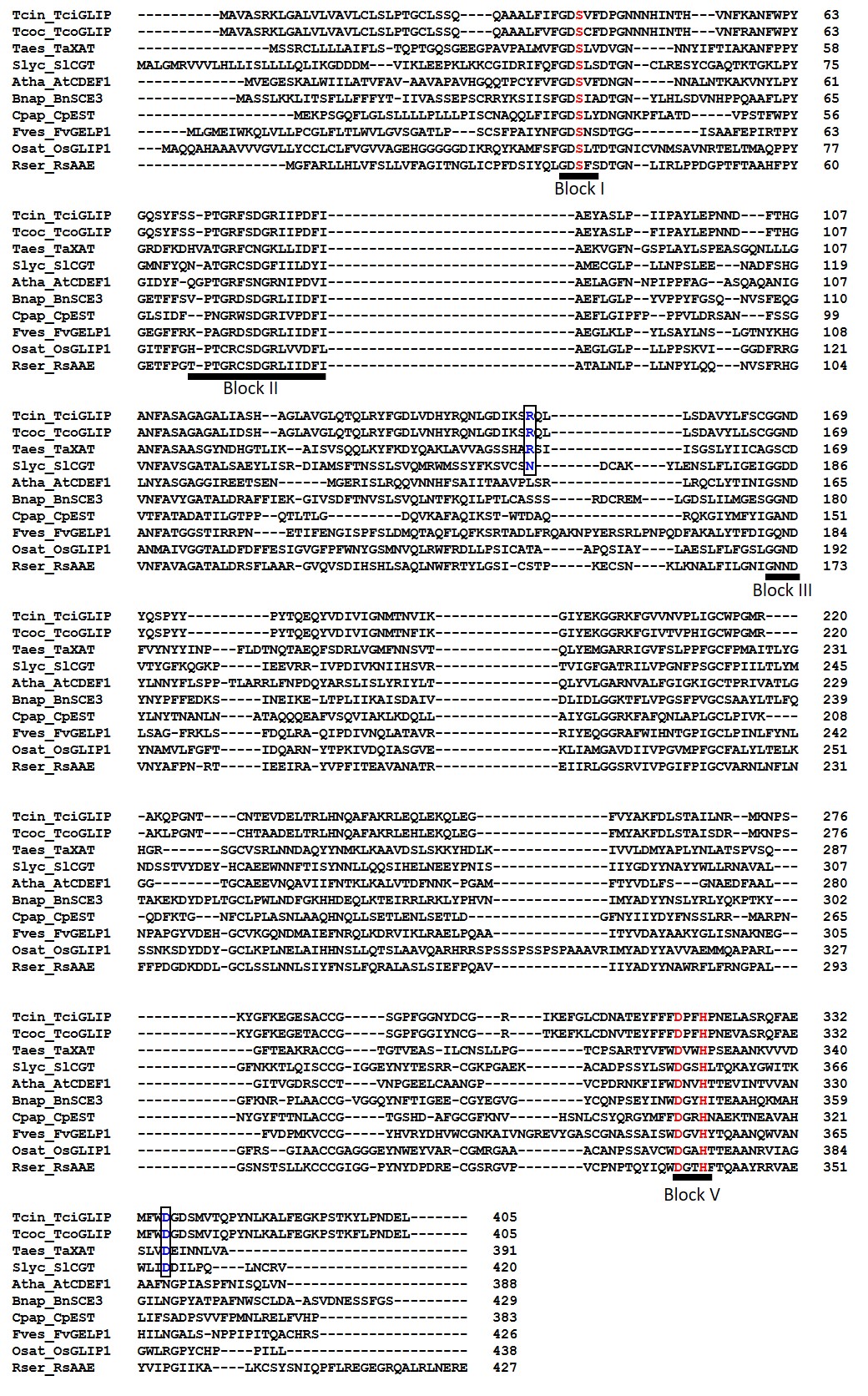

Supplement: Supplementary file 1 [file ijms-23-15141-s001.zip › Supplemental Information S1.jpg]

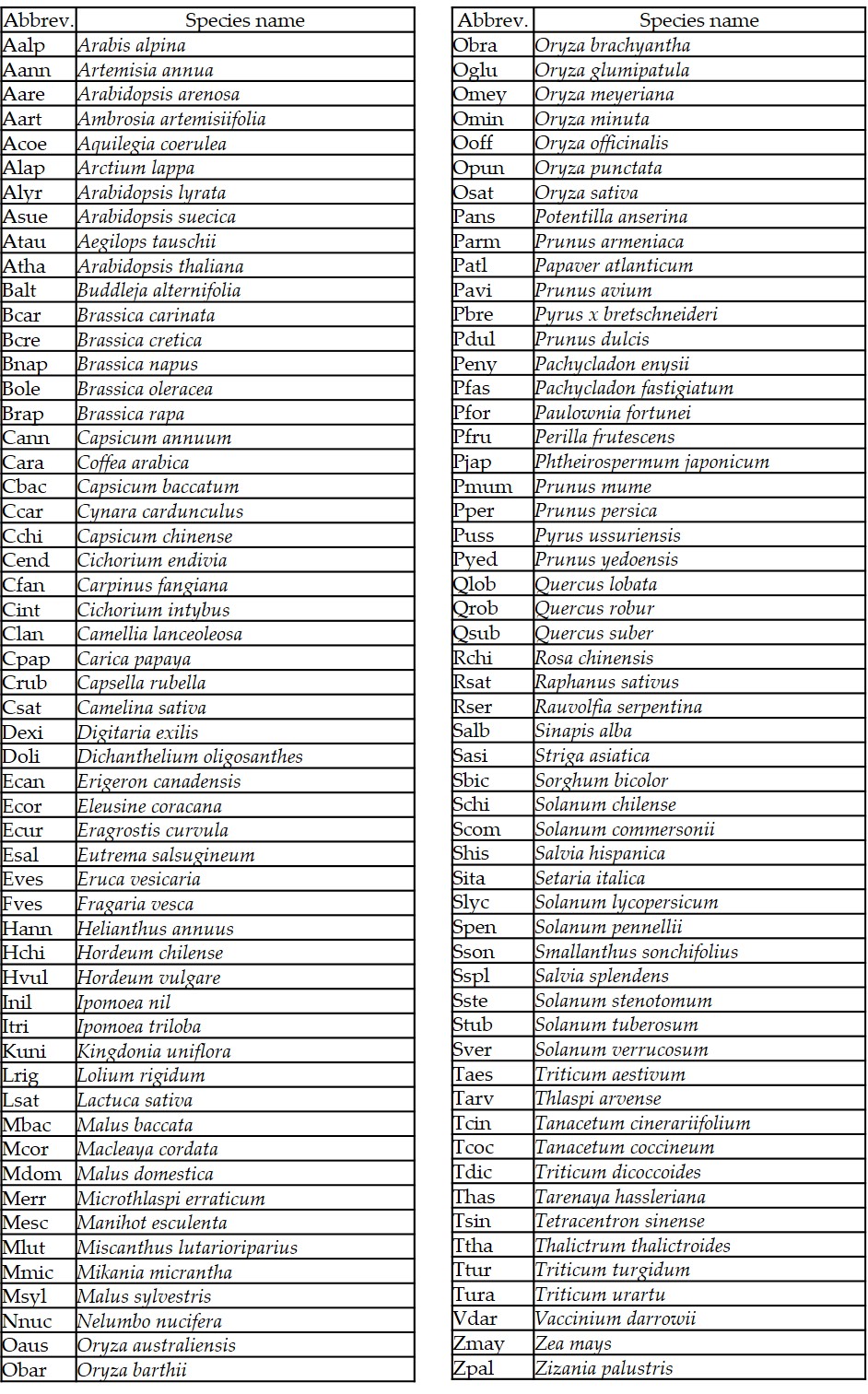

Supplement: Supplementary file 1 [file ijms-23-15141-s001.zip › Supplemental Information S2.jpg]
